# Current Status of the Bioactive Properties of Royal Jelly: A Comprehensive Review with a Focus on Its Anticancer, Anti-Inflammatory, and Antioxidant Effects

**DOI:** 10.3390/molecules28031510

**Published:** 2023-02-03

**Authors:** Sara Botezan, Gabriela-Maria Baci, Lilla Bagameri, Claudia Pașca, Daniel Severus Dezmirean

**Affiliations:** Faculty of Animal Science and Biotechnology, University of Animal Sciences and Veterinary Medicine Cluj-Napoca, 400372 Cluj-Napoca, Romania

**Keywords:** apitherapy, royal jelly, MRJPs, 10-HDA, anticancer, anti-inflammatory, antioxidant, bioactive components, biological functions

## Abstract

Royal jelly (RJ) has been one of the most widely used natural products in alternative medicine for centuries. Being produced by both hypopharyngeal and mandibular glands, RJ exhibits an extraordinary complexity in terms of its composition, including proteins, lipids, carbohydrates, polyphenols, vitamins, and hormones. Due to its heterogeneous structure, RJ displays various functional roles for honeybees, including being involved in nutrition, learning, memory, and social behavior. Furthermore, a wide range of studies reported its therapeutic properties, including anticancer, anti-inflammatory, and antioxidant activities, to name a few. In this direction, there is a wide range of health-related problems for which the medical area specialists and researchers are continuously trying to find a cure, such as cancer, atherosclerosis, or infertility. For the mentioned diseases and more, it has been proven that RJ is a key player in finding a valuable treatment. In this review, the great impact of RJ as an alternative medicine agent is highlighted, with a focus on its anticancer, anti-inflammatory, and antioxidant activities. Moreover, we link it to its apitherapeutic potential by discussing its composition. Herein, we discuss a wide range of novel studies and present the latest research work.

## 1. Introduction

Since ancient times, mankind has taken advantage of the benefits associated with beehive products, such as honey, propolis, bee pollen, beeswax, bee venom, and RJ, and distinct cultures have been using them to treat and prevent diseases [1]. Because of their specific biochemical profile, all of these bee-derived products are highly bioavailable [2].

RJ, also known as Apilak or Queen Bee Jelly, is a yellowish-white, creamy substance secreted in the hypopharyngeal and mandibular glands of worker bees. It is the only substance fed to the queen larvae and the worker bee larvae are also fed with RJ, but only in the first three days, then a mixture of honey, pollen, and nectar, also called bee bread, constitutes the main diet of the latter [3]. Through its nutrient-rich chemical composition, RJ represents an important source of food for the bee family, playing an essential role in the biology of these insects. For humans, RJ represents a very important nutraceutical, functional food, and nutritional supplement [4] that can efficiently complement a healthy diet. Furthermore, it can also be useful in the diets of various animal species [5].

Ever since the 1980s, Chinese researchers and beekeepers have been implementing genetic selection strategies to improve RJ production. Starting with Italian bees, a strain of RJ-producing bees (also known as *Apis mellifera ligustica*) has been obtained through this selection, yielding up to 10 times more RJ than Italian bees. Thus, China is currently producing over 90% of the global RJ output, with a whopping 3500 tons per year and an annual market worth of approximately USD 40 million [6].

Currently, RJ is being popularized among apitherapy societies, having the potential to be incorporated into various food products, supplements, and medicines. Experts in the field have taken on the mission of promoting this product and disseminating as much information as possible about it to the public [7,8]. Moreover, as healthy and conscious lifestyle choices gained popularity among consumers in recent years, the demand for natural foods has risen quickly. Apiculture products have become increasingly popular due to their essential nutritional and medicinal benefits [9].

The use of RJ has both advantages and disadvantages. The main advantage is that RJ represents a natural product with a complex, nutrient-rich chemical composition. Moreover, RJ can be obtained through established, specific beekeeping techniques. The disadvantages include: the adulteration of RJ, the fact that it is perishable, its production is seasonal, and its price is high [4,10,11,12,13,14].

Importantly, RJ possesses an impressive array of bioactive compounds, which include, but are not limited to: major royal jelly proteins (MRJPs), 10-hydroxy-2-decenoic acid (10-HDA), and hormones. These biologically active components are thought to be responsible for RJ’s beneficial properties, individually or synergistically. Among other effects, RJ possesses anticancer, anti-inflammatory, and antioxidant effects. Studies have been carried out on cells, laboratory animals, and humans. However, clinical trials are found in a smaller number. RJ has numerous other health-promoting activities: antimicrobial, antibacterial, immunoregulatory, antidiabetic, improving reproductive health (through its estrogen-like activity, and it contains testosterone, so it can be beneficial for men as well), wound-healing, lifespan-prolonging, antiaging, antilipidemic, anti-hypertensive, antiviral, antiparasitic, organo-protective (neuroprotective, hepatoprotective, etc.), neuromodulatory, cell-growth promoting activity (on normal, healthy cells), obesity-restraining, and memory-improving [3,15,16].

There are a lot of health problems that must be cured by medical doctors and researchers, such as cancer or diseases connected to an abnormal inflammatory process or antioxidant activity of the organism. Cancer, auto-immune diseases, and dysfunctions, associated with oxidative stress, are devastating conditions with a high prevalence worldwide. In regard to cancer alone, despite recent advances in screening techniques and treatment options, early diagnosis is challenging because the prognosis is poor and the survival rates remain low [17].

**Figure 1:**
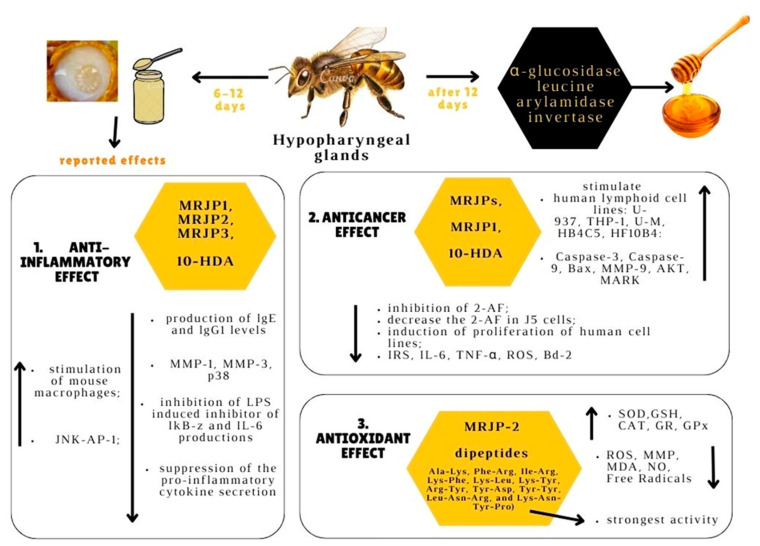
Anticancer, anti-inflammatory, and antioxidant effects of RJ and their mechanisms. RJ is produced by hypopharyngeal glands of bees with ages between 6–12 days. This bee product can be used for its effects: 1. *Anti-inflammatory effect*: increase in c-Jun N-terminal kinases-activating protein-1 (JNK-AP-1) and the stimulation of mouse macrophages; inhibition of lipopolysaccharide (LPS)-induced inhibitor of kappa-B-zeta (IkB-z) and IL-6 productions, inhibition the production of lgE and immunoglobulin lgG1 levels, inhibition of the activities of MMP-1, MMP-3, p38. 2. *Anticancer effect:* stimulation of growth of the following human lymphoid cell lines: U-937, THP-1, U-M, HB4C5, HF10B4; increase in the activity of MMP-9 (matrix metallopeptidases-9), AKT (protein kinase B) and MAPK (mitogen-activated protein kinase); inhibition the metabolism of 2-aminofluorene (2-AF) metabolites in a human liver tumor cell line and decrease the 2-AF in J5 cells; induction of proliferation of human cell lines; decrease in IRS (insulin receptor substrate 1) activity; IL-4 (interleukin-4); TNF-α(tumor necrosis factor); ROS (reactive oxygen species). 3. *Antioxidant effect:* increase in SOD (superoxide dismutase), GSH (glutathione); CAT (catalase); GR (glutathione reductase), GPx (glutathione peroxidase) activities; and decrease in the levels of ROS (reactive oxygen species); MMP (matrix metallopeptidases), MDA (malondialdehyde), NO (nitric oxide), and free radicals (Created with canva.com, accessed on 17 January 2023).

Being a natural beehive product, RJ is a powerful antioxidant [18], anticancer [19], and anti-inflammatory [20] agent. Considering these properties of RJ and its usefulness in apitherapy, the present review is aimed at discussing groundwork, as well as new research, including in vitro studies, animal studies, and a few clinical trials in this vast and continuously developing domain. In other words, the present paper is aimed at highlighting the apitherapeutic potential of RJ, with a special focus on antitumor, anti-inflammatory, and antioxidant roles. RJ’s chemical composition and the main bioactive compounds in RJ, namely MRJPs, 10-HDA, and RJ hormones, were presented. The main part of this work consists of a synthesis of the specialty literature on the three abovementioned functional properties of RJ. Lastly, three apitherapeutic applications of RJ, namely: wound healing, its role as a nutraceutical, as well as its role in aging and longevity, were briefly discussed. Even if reviews have already been written on these topics, we attempted to include the most recent and comprehensive information in the field and bring a fresh perspective on the subject. Figure 1 represents an illustration of the three therapeutic effects of RJ that were discussed in the present review and the mechanisms behind them.

## 2. Royal Jelly: Chemical Characterization, Quantitative Determination, and Storage

### 2.1. General Remarks

RJ represents a rich source of nutrients and bioactive compounds which depends on different factors such as beekeeping season, the geographical orientation of the apiary, chemicals used, meteorological conditions, the ecosystem where the honeybees live, and the plant cultures that the insects have access to. Another parameter that can influence the chemical composition is the race and caste of the honeybees, physiological and metabolic differences between the nurse bees, and the harvest time of RJ [21]. This bee product is a functional food that drives the phenotypic development of the female bee larvae, allowing its transformation into a fertile bee queen instead of a sterile worker bee [22], additionally playing a crucial role in bee brain functions, such as memory, learning, and social behavior [23]. At the same time, RJ can be consumed by humans as a functional food, and it holds a high commercial value for its nutritional and nutraceutical properties [24].

The chemical composition of RJ consists of water (60–78%), proteins (9–18%), carbohydrates (7–18%), lipids (3–8%), mineral salts (0.8–3%), small amounts of polyphenols, vitamins, and enzymes [25,26]. In general, RJ is relatively acidic with a high buffering capacity (pH 3.20–4.01) and the total acidity varies between 2.48–4.66 mL 0.1 N NaOH/g [27]. Sugar composition, moisture, protein, and 10-HDA contents are the most prevalent criteria in the determination of the quality and authenticity of RJ [26,28].

### 2.2. Sugar Composition

Sugars represent about 30% of the dry matter in RJ [4,29,30]. In general, the main carbohydrates found in RJ are monosaccharides (fructose and glucose) [31] representing 90% of the total sugar fraction of RJ, and sucrose accounts for 0.8–3.6% [32]. RJ contains very small amounts of other sugars such as maltose, trehalose, melibiose, ribose, and erlose. Some authors stated that the determination of sugars could provide important information about RJ’s quality, including the detection of possible adulteration with honey or sugars [30]. Adulteration can also be achieved using corn starch, milk, yoghurt, or egg white [4].

### 2.3. Proteins, Peptides, and Amino Acids

Proteins, including minor RJ proteins and MRJPs are present in RJ with an average of 9–18% [30,31] and in the International standards ISO 12824 between 11–18%. A total of 80% percent of RJ proteins are represented by the so-called MRJPs, which are playing specific physiological roles in honeybee queen development and include numerous essential amino acids. The MRJP family includes nine members, specifically MRJP1, MRJP2, MRJP3, MRJP4, MRJP5, MRJP6, MRJP7, MRJP8, and MRJP9 [23,30].

Like proteins, peptides represent specific sequences of amino acids in RJ that have biological activities and potential health applications [33]. Among the peptides in RJ, there are: apisimin and jelleines I, II, III, and IV [4]. Royalisin is another peptide from RJ and it was found to have potent antibacterial activity against Gram-positive bacteria, such as *Clostridium*, at low concentrations [34,35].

RJ is rich in amino acids, including lysine, proline, cysteine, aspartic acid, valine, glutamic acid, serine, glycine, cysteine, threonine, alanine, tyrosine, phenylalanine, hydroxyproline, leucine, isoleucine, and glutamine. These high amounts of amino acids in the MRJP family are essential for the development of both queen bees and larvae [36].

### 2.4. Lipids and Fatty Acids

Among the main nutritional elements of RJ, lipids constitute 7–18% of RJ content; 90% of these lipids are unique short hydroxy fatty acids with 8–12 carbon atoms in their chain and dicarboxylic acids [32]. The most abundant RJ fatty acids are 32% 10-HDA, 22% 10-hydroxy-*trans*-2-decenoic acid (10-H2DA), 24% gluconic acid, 5% dicarboxylic acids [33]. Other fatty acids found in RJ are: 10-hydroxydecanoic acid (10-HDAA), 8-hydroxy octanoic acid, 3-hydroxydecanoic acid, 3,10-dihydroxydecanoic acid, 9-hydroxy-2-decenoic acid, 1,10-decanedioic (sebacic) acid, and 2-decenedioic acid [4]. The most important parameter for quality control of RJ is 10-HDA, being an indicator of freshness, quality and authenticity and it is a fatty acid that is present only in RJ [27,37,38].

### 2.5. Minerals, Flavonoids, Vitamins, and Other Components

RJ contains small amounts (1.5%) of various minerals and trace elements such as K, Na, Mg, Ca, P, S, Cu, Fe, Zn, Al, Ba, Sr, Bi, Cd, Hg, Pb, Sn, Te, Tl, W, Sb, Cr, Mn, Ni, Ti, V, Co, and Mo. Whereas concentrations of trace and mineral elements in RJ vary according to the botanical origin, they are generally constant [7,31]. The main element is potassium (2462–3120 mg/kg) [4].

The proportion of phenolic components in RJ is 23.3 ± 0.92 gallic acid equivalent (GAE) g/mg; however, these values can change depending on the dietary source. The total flavonoid content in RJ is 1.28 ± 0.09 rutin equivalent (RE) g/mg 32]. Flavonoids are the largest and most important group of phenolic compounds [18]. According to the level of structural complexity, flavonoids of RJ can be differentiated into four groups: (1) flavanones, e.g., hesperetin, isosakuranetin, and naringenin; (2) flavones, e.g., acacetin, apigenin and its glucoside, chrysin, and luteolin glucoside; (3) flavonols, e.g., isorhamnetin and kaempferol glucosides; and (4) isoflavonoids, e.g., coumestrol, formononetin, and genistein [32].

RJ contains small amounts of various B-group vitamins (B1, B2, B6, B8, B9, and B12), ascorbic acid (vitamin C), vitamin E, and vitamin A [31]. Pantothenic acid (vitamin B5) is the most abundant vitamin in RJ (52.8 mg/100 g), followed by niacin (42.42 mg/100 g) [9].

In addition, the interest substance contains other biochemicals, namely adenosine monophosphate (AMP) and its oxide adenosine monophosphate N1 (AMPN1) oxide, acetylcholine, hormones (prolactin, testosterone, estradiol) [3], as well as organic acids and their esters: octanoic acid, benzoic acid, 2-hexenedioic acid and its esters, dodecanoic acid and its ester, known as 1,2-benzenedicarboxylic acid [18]. Moreover, enzymes such as glucose oxidase, alpha-glucosidase and gluco-cerebrosidase are also found in the food of the queens. All these additional components are found in very small amounts [4]. The nutritional requirements for RJ are listed in Table 1.

The chemical composition (Table 2) of RJ varies according to different factors, such as honeybee races, floral origin, age of bee larvae and beekeeping practices. There is a regulation of the national and international standards and also in different studies, stating the levels for the quality assurance parameters of fresh RJ. Although RJ is internationally traded for its beneficial properties in the food, cosmetic, and pharmaceuticals industries, there is a major need to establish and adopt consistent quality parameters and standards within producing countries [39], the same as in Argentina, Bulgaria, Turkey, Brazil, Japan, China, and Switzerland.

### 2.6. Conditions for Maintaining RJ Quality

Temperature is an important factor affecting the physicochemical properties of RJ during its maintenance. For this reason, researchers established the storage and shelf life of RJ depending on this parameter. Kausar and More [44] made a comparison between the organoleptic features and the physicochemical properties of fresh and lyophilized RJ, to prove that the latter can also exert the same health benefits as the first. The authors concluded that when lyophilized, RJ is similar in composition to fresh RJ and freeze-drying does not degrade the substance of interest. Therefore, RJ lyophilization can be a useful strategy that enables the storage time to be increased and the RJ to be stored at room temperature, while still maintaining its quality.

Maghsoudlou et al. [12] described the storage stability and shelf life of RJ. Hence, the shelf life for fresh RJ ranges between 6 months (4 °C)–2 years (<−18 °C) and lyophilized RJ can be used for up to 1 year (4 °C) and more than 2 years (<−18 °C). At the same time, RJ can be used in other products (such as honey) and the shelf life in this case is 2 years at room temperature.

## 3. Main Bioactive Compounds in Royal Jelly

### 3.1. Major Royal Jelly Proteins (MRJPs) Family

In terms of RJ’s soluble protein content, over 80% is represented by MRJPs. Containing a wide range of essential amino acids, such as arginine, histidine, or lysine, these proteins play a key role in the process of honeybee queen’s development. It is well documented that for each of the target proteins, MRJP 1-9, the honeybee’s genome harbors a distinct gene [14], which are located on chromosome 11 [45]. These proteins exhibit a high amino acid homology of up to 70% [3]. In addition, it has been reported that the Yellow proteins are found in *Drosophila* and other insects, but also in some bacteria and share MRJP’s evolutionary origin [46,47]. Furthermore, it has been shown that the MRJPs evolved from the *yellow-e3* gene, which exhibits a key role in early bee brain development [48]. The MRJPs are synthesized in honeybee cephalic glands, hypopharyngeal and mandibular; each of the nine members was identified in RJ.

#### 3.1.1. MRJP1

Among the members of this protein family, the dominant glycoprotein in RJ is MRJP1, reaching up to 66% of the total amount of MRJPs [14,33,47]. MRJP1′s architecture involves a chain of 413 amino acids, mainly valine and leucine, [16,33,49] and its secondary structure mainly implies β-sheets (38.3%), followed by 20% β-turns, and 9.6% α-helices [32]. In RJ this specific glycoprotein is found in a monomeric state, royalactin (55 kDa), but also in an oligomeric form, apisin (280–450 kDa) [16,47]. Royalactin increases body and ovary size, and triggers physiological changes that play an important role in the queen bee differentiation. The second-mentioned state represents a complex formed by royalactin, 24-methylenecholesterol and apisimin, a serine-valine-rich protein [16]. The MRJP1 oligomer exhibits great biochemical heat resistance, but also long storage stability compared to royalactin [47,49]. It creates a pH-dependent fibrillary network, which achieves the necessary viscosity to this bee product, stopping the larvae from falling from the queen cells [50,51]. Moriyama et al. (2015) [49] investigated the MRJP1 heat-resistant features by exposing the glycoprotein to 56, 65, and 96 °C; more specifically, the proliferative activity was assessed. The results showed that until 56 °C, the oligomer’s molecular state was maintained. On the other hand, at 65 °C and 96 °C, the molecular forms transformed in macromolecular aggregates. Interestingly, even if the growth activity was attenuated at 96 °C, the cell proliferation activity was not impacted.

The *mrjp1* gene consists of 3038 bp and includes six exons and five introns [52], and it has been reported that *mrpj1* is expressed in prominent structures of the bee brain, suggesting that MRJPs’ role is not limited to nutrition, but these proteins are also involved in bees’ social behavior, memory, and learning [47,53]. In this direction, tremendous effort is being made to perform functional analyses on target genes, such as *mrjp1*. For instance, Kohno et al. (2016) [54] used the clustered regularly interspaced short palindromic repeats (CRISPR)-cas9 system to knock out the *mrjp1* gene in order to investigate its dispensable status in the honeybee, *Apis mellifera*. The authors used a Cas9 expression vector and designed a single-guide RNA (sgRNA) based on a target sequence of 20-base, and an NGG sequence was added following the sgRNA, as protospacer adjacent motif. After the eggs were laid, for up to three hours, the injection was performed into the egg’s dorsal posterior side. By injecting 57 fertilized embryos, they obtained six queens, and two of them laid eggs under laboratory conditions. From two queens, one of them led to genome-manipulated drones, and 20 drones of the total of 161 produced, were genome-edited (12.4% efficiency). Their results revealed that the *mrjp1* gene exhibits no impact on normal drone development, at least until the pupal phase.

Although MRJP1 is the pivotal protein of this group, the other members also play crucial roles for honeybees (Table 3). The post-translational modifications, such as phosphorylation, N-glycosylation, or methylation, directly impact the function of each MRJP.

#### 3.1.2. Therapeutic Impact of MRJPs

Among the crucial roles that RJ exhibits for the honeybee, a wide range of researchers across the world demonstrated its beneficial effects for human health, including its antitumor, antiviral, anti-inflammatory, antioxidant, or antimicrobial activities. Even if the most important member of MRJP’s family is MRJP1, numerous studies reported the therapeutic value of other members of the MRJP family and underlined its role as key active components [16]. There is a wide range of novel studies that reported the MRJPs’ beneficial role for human health [53,58,61,62,63,64,65,66,67].

In terms of its pharmacological effects on human health, MRJP1 has a wide range of pharmacological properties, including wound healing, antibacterial, antitumor, antihypertensive, antifungal activities, hypocholesterolemic effects, immune enhancement, as well as cell growth-promoting activities. In studies involving rat hepatocytes, MRJP1 was found to promote cell proliferation and induce albumin synthesis even in the absence of fetal bovine serum, potentially by functioning as biosimilars [22,68]. In mouse macrophages, MRJP2 promoted the release of the anticancer compound tumor necrosis factor-α (TNF-α) [69]. Moreover, through the inhibition of TNF-α, intracellular reactive species, and mixed lineage kinase domain-like protein, MRJP2 might alleviate hepatic necrosis against carbon tetrachloride-induced hepatotoxicity. MRJP2 could be a trustworthy treatment strategy for hepatic disorders. Additionally, recombinant MRJP2 and MRJP4 can kill various microorganisms by adhering to the cell walls of fungus, yeast, and bacteria, causing cell wall destruction [70].

As MRJP3 binds to and stabilizes ribonucleic acid (RNA), it is hypothesized that bees are able to transmit RNA across individuals, through secretion and consumption. It is also believed that this RNA transfer from worker bees to larvae may promote social immunity against diseases [48]. Kohno et al. (2004) discovered that MRJP3 impacts the immunological responses of T-cells by downregulating the production of interleukin (IL)-4, IL-2, and interferon (IFN) in research on the health aspects of MRJP3. Moreover, they reported that MRJP3 acts as an anti-allergic agent by reducing immunoglobulin (Ig)E and IgG1 synthesis. According to the authors, this protein inhibits the production of pro-inflammatory cytokines, such as TNF-, IL-6, and IL-1, in activated mouse macrophages, acting as an anti-inflammatory drug both in vivo and in vitro [54].

#### 3.1.3. Purification of MRJPs

Regarding MRJP purification approaches, chromatography is the primary technique used for protein purification, based on extracting the substances of interest by using the interactions between a mobile and stationary phase. Using chromatographic procedures, molecules are separated from mixtures based on their characteristics, such as size, hydrophobicity, electric charge, and affinity to ligands. Among chromatographic methods, ion exchange (adsorption), hydrophobic interaction, affinity, molecular exclusion (gel filtering) are the most used for protein purification. MRJP1, MRJP2, MRJP3, and MRJP5 have a positive electrical charge due to RJ’s natural pH value of about 4.0, having isoelectric points (pI) between 5.03 and 6.65 [71].

### 3.2. 10-Hydroxy-2-Decenoic Acid (10-HDA)

More than 50% of the free fatty acid content of RJ is represented by 10-HDA, which has not been found in any other natural product, not even in another bee-related product [27]. Several studies have demonstrated that 10-HDA has a wide range of health-promoting activities, including immunoregulatory [3], anti-inflammatory, antioxidant [27], antidiabetic, antibacterial [72], and antitumor [33,72]. Four unsaturated fatty acids (10-H2DA, 10-HDAA, *trans*-2-decenoic acid, and 24-methylenecholesterol) found in RJ were demonstrated to have estrogen receptor (ER)-binding activity [73]. The interaction of these substances with ERs resulted in changes in gene expression and cell growth [74]. Recent research revealed that its antitumor properties have not been precisely outlined. However, Lin et al. (2020) [73] investigated the mechanisms behind 10-HDA’s action in A549 human lung cancer cells. According to their findings, 10-HDA exhibited no harmful effects on healthy cells while inhibiting the growth of three human lung cancer cell lines, namely A549, NCI-H23, and NCI-H460.

A study conducted by Albalawi et al. (2021) [75] aimed to examine the antitumor effect of this natural product, alone and in combination with cyclophosphamide (CP), an alkylating drug frequently used in neoplastic tumor treatment. Mice with *Ehrlich* solid tumors were enrolled in this research, all of them treated with 10-HDA (2.5 and 5 mg/kg) alone and combined with CP (25 mg/kg), once a day, during a 2-week period. However, more research, particularly in a clinical context, is necessary to validate these findings, the fatty acid at the dosages of 2.5 and 5 mg/kg, especially in conjunction with CP, exhibited potential antitumor effects against EST in mice.

Peng et al. (2017) [76] investigated the anti-melanogenic and depigmenting activity of 10-HDA. The skin whitening effect was monitored by applying 0.5%, 1%, and 2% of 10-HDA-based cream on mouse skin (C57BL/6 J strain) for a period of three weeks. 10-HDA, even at a dosage of 0.5%, was able to considerably stimulate skin whitening. Moreover, 10-HDA decreased the tyrosinase activity, along with the production of melanogenic enzymes, by inhibiting the microphthalmia-associated transcription factor (MITF) protein expression in B16F10 melanoma cells. These findings showed that this fatty acid has the potential to serve as a natural, effective, and safe melanogenesis inhibitor in the health and cosmetic industry.

In vitro anti-inflammatory effects of 10-HDA in lipoteichoic acid (LTA) from *Staphylococcus aureus* stimulated RAW 264.7 cells were evaluated by Chen et al. (2018) [77]. They observed a decrease in the number of important inflammatory genes, such as IL-1, IL-6, cyclooxygenase-2 (COX-2), and monocyte chemoattractant protein-1 (MCP-1). Furthermore, 10-HDA’s impact on lung damage caused by LTA was tested on mice. Results showed that 100 mg/kg of 10-HDA can have protective properties by controlling the release of inflammatory cytokines including IL-10, MCP-1, and TNF-α. They provide evidence that this fatty acid had significant, dose-dependent inhibitory effects. The results demonstrate 10-HDA’s powerful anti-inflammatory effects; however, authors suggest further research to fully understand this fatty acid’s mechanisms of action.

### 3.3. Hormones

As for the hormone content of RJ, it contains testosterone, progesterone, prolactin, estrogen [15], and estradiol [3]. Due to its resemblance to estrogens, RJ is widely used by women in order to relieve and treat menopause, as well as aging-related diseases. Clinical trials demonstrated that oral administration of 1g of RJ per day has a decreasing effect on the severity of the premenstrual syndrome. Moreover, by using this natural substance, a life quality improvement was observed amongst the reproductive-aged women [78].

RJ is supposed to play an important role in the hormonal balance, by increasing testosterone production, along with the synthesis of estrogen. 10-HDA improves hormonal balance by increasing the production of ovulation hormones and preventing the depletion of the follicular pool [79].

Other findings indicate that, when given to adult male rabbits at a dose of 150 mg/kg body weight, RJ has a beneficial impact on libido, glucose, blood testosterone concentration, total proteins, fertility, as well as sperm production and quality. It was found that RJ supplementation can enhance the physiological state of heat-stressed rabbits and it can also prevent summer infertility [80]. However, more studies on RJ hormones are needed to better understand their way of action.

## 4. Functional Properties of Royal Jelly

### 4.1. Anticancer Role

Cancer is a general term used to describe an extensive palette of diseases that can affect any part of the organism. The main characteristic of this disease is the rapid generation of abnormal cells, that divide uncontrollably and exceed their usual boundaries, forming malignant tumors. Eventually, cancerous cells can spread and invade neighboring tissues and organs in a process called metastasis, which eventually leads to death. The origin of this disorder resides in the accumulation of genetic alterations in somatic cells. These mutations have the capacity to modify the normal phenotype of cells, granting them the possibility to escape homeostatic regulation that maintains normal cell numbers [81]. In short, abnormal cell growth, division, and survival stand at the root of all cancers.

In order to divide, cells need to copy their deoxyribonucleic acid (DNA) and then pass it to the daughter cells. While this process is fairly accurate, the fidelity is not perfect. Thus, if there are any mutations that appear during DNA replication and are not repaired in time, they can be passed to the descendant cells and carried by all subsequent generations. Therefore, the probability that new mutations are introduced at every division is quite high. Billions of cell divisions corroborated with flawed DNA copying renders intratumor heterogeneity inevitable [81]. Therefore, cancer has such a complex pathogenesis and pathophysiology, being a disorder that involves dynamic genomic changes [82].

The instructions for life are all written in the DNA, which dictates the fate of cells. In a normal state, all physiological processes and complex mechanisms that underlie viability are tightly regulated and homeostasis is maintained. Nonetheless, if certain genes are affected by deleterious mutations, the malignant transformation of cells may arise. Cancer progression is known to be driven by modifications of gene expression patterns and the accumulation of various genetic mutations [83] over a long period of time. Thus, cancer develops slowly and there can be an interval of even 20 years from carcinogen exposure to clinical detection for most human solid tumors [84].

Back in 2000, Hanahan and Weinberg came in support of the idea that the complexities of the disease could be simply described by several underlying principles, that would later be known as the hallmarks of cancer. Describing them in detail is beyond the scope of this work, therefore, they will be listed as follows: resisting cell death, enabling replicative immortality, sustaining proliferative signaling, inducing angiogenesis, activating invasion and metastasis, and evading growth suppressors. Eleven years later, in the continuation of the article, known as “Hallmarks of cancer: the next generation”, the authors added two more features that define malignant cells: evading immune destruction and reprogramming of energy metabolism [85].

Over the years, scientists alongside medical specialists have developed several strategies that are still used today in the fight against cancer. These are: surgery, chemotherapy, and radiotherapy. Among the relatively new cancer therapies, molecular targeted therapies and immunotherapy are included. However, most of these approaches have significant limitations and cancer is oftentimes diagnosed at a stage that is too advanced for the patient’s life to be saved [4,14]. In this context, it is imperative to elaborate more effective, faster-acting treatments and to lessen their side-effects, given the health risks and high mortality rates of cancer. One particular way of decreasing these health-detrimental consequences and inhibiting tumor growth is to use natural anticancer substances since these are easy to obtain and are reasonably safe [4,19]. The fact that certain bee-derived products can help suppress cancers is commonly known. More specifically, RJ’s antitumor property has been researched and mechanisms such as cancer-cell multiplication, metastasis and tumorigenesis repression have been found and attributed to this bee secretion. These mechanisms are due to the restriction of angiogenesis and the ability of RJ to activate the immune system [78,86].

Therefore, several studies have demonstrated that RJ can exert an antitumor action and can represent a complementary therapy in malignancies [78]. For instance, Bincoletto et al. (2005) [87] have pointed out that RJ can produce a decrease in prostaglandin E2, which may result in the suppression of the spleen’s hematopoietic activity and the improvement of myelosuppression in Ehrlich tumor mice, thus improving the animal’s survival chances [3]. In another research performed by Nakaya et al. (2007) [88], RJ has been proven to suppress MCF-7 human breast cancer cell multiplication, which was triggered by bisphenol A, an environmental estrogen. Wang and Chen (2019) [89] unveiled that the proliferation of a leukemia cell line U937 can be significantly reduced by different types of treated RJ extracts. This can happen because cytokine release by mononuclear cells is prompted by the aforementioned RJ extracts. Caspase-dependent apoptosis is triggered in HepG2 human hepatoma cells by MRJP2 and X1, which is this protein’s isoform [90]. Using a murine breast cancer model, Zhang et al. (2017) [91] showed that RJ therapy decreased tumor mass and improved the antioxidative activity of several organs, including the kidneys and liver. Additionally, the treatment stimulated antioxidant enzymatic activity, highlighting a possible link between RJ’s antitumor and antioxidant bioactive properties.

Abandansari et al. (2018) [92], revealed that the combined treatment of doxorubicin and RJ managed to intensify the cytotoxic effect on prostate cancer cell line PC3. Thus, RJ complemented doxorubicin’s antitumor activity, making it possible to lower the dose of the drug in future clinical trials and thus, to ease its side effects in patients. Furthermore, another group of researchers underlined that RJ therapy on patients reduced the frequency of negative side effects in the case of tyrosine kinase inhibitor treatment of renal cell carcinoma, in the context of two randomized, double-blinded clinical trials. Consequently, RJ intake resulted in an improved quality of life and lowered the toxicity induced by the tyrosine kinase inhibitor treatment [93,94]. Moreover, RJ is deemed to display a shielding effect against classical cancer therapy-induced cytotoxicity [19].

Several components that may be responsible for the antitumor activity of RJ are flavonoids, namely naringenin, acacetin, apigenin, chrysin, genistein, coumestrol, luteolin, hesperetin, formononetin, kaempferol, glycoside, and isosakuranetin [95]. Repression of malignant cell multiplication, stimulation of apoptosis, regulation of antioxidant enzyme activities, autophagy, and cell cycle suppression are only a few of the mechanisms through which flavonoids from RJ demonstrate their anticancer effects [96,97]. Polyphenols in RJ, exhibit their anticancer effects through a variety of mechanisms, including cell death through altered signaling pathways, cell cycle arrest, and activation of apoptosis, along with their antimetastatic and antiangiogenic properties [97,98].

Regarding 10-HDA, two in vitro experiments have been conducted that suggest its antitumor effect. These underline that RJ exerts this effect by modulating oxidative stress and inflammation in colon cancer cells [48,99]. Thus, the abovementioned studies highlight a link between anticancer, anti-inflammatory, and antioxidant properties possessed by the food of the queens. Nevertheless, new studies are needed on this topic, with more comprehensive investigations in different cancers, in order to validate this possible effect of 10-HDA, as well as to unravel its therapeutic limitations [19]. Furthermore, a derivative of this fatty acid, called 4-hydroperoxy-2-decenoic acid ethyl ester (HPO-DAEE), can drive human lung cancer cell line A549 to go into apoptosis via two cellular signaling pathways, namely CCAAT-enhancer-binding protein homologous protein (CHOP) and ROS-ERK-p38 [100].

Even though many studies have found beneficial effects of RJ and its components on malignancies, the mechanisms underlying these effects are not yet fully understood. In order to achieve this comprehension, research on the molecular changes that RJ generates on various factors related to cancer (such as cellular stress generated by reactive oxygen species (ROS), cell survival, and inflammatory processes) is needed. In this regard, RJ demonstrated its anticancer properties by inducing apoptosis, which increased antioxidant factor activity, inhibited raised serum indicators, and histological modifications, and controlled inflammatory factors, among other mechanisms [19].

### 4.2. Anti-Inflammatory Activity

The first reaction of the organism to injury or infection is represented by inflammation. This rather complex process triggers various physiological immunological pathways. However, when inflammation is dysregulated due to certain factors, it can damage the adjacent tissue and lead to a variety of pathologies. Inappropriate inflammation is associated with the release of inflammatory cytokines, (ROS, eicosanoids derived from arachidonic acid and specific cellular adhesion molecules [4,101]. In a link to the previous chapter, cancer is a life-threatening disease that is strongly associated with abnormal inflammatory processes. RJ has exhibited anti-inflammatory properties in a variety of illnesses connected to an aberrant inflammation process [4]. For example, a group of researchers from China focused on investigating the anti-inflammatory activity of RJ on the BV-2 murine microglial cell line treated with lipopolysaccharides (LPS), which are known to trigger inflammation. The researchers found that RJ had a protective effect on the cells and alleviated the inflammatory response, the underlying mechanisms could be related to the synthesis of pro-inflammatory cytokines being blocked. Pro-inflammatory protein COX-2 expression levels could also be significantly inhibited by RJ. Additionally, RJ inhibited the nuclear factor kappa B (NF-kB) and c-Jun N-terminal kinase (JNK) pathways, lowering the rate of inflammatory mediators synthesis through these mechanisms [102].

In regard to research conducted on human subjects, a recent study involved the treatment of asymptomatic overweight patients with 333 mg RJ per day for 8 weeks. It was discovered that this treatment had anti-inflammatory effects on the patients, producing a decrease in the level of IL-6 and the inflammatory marker c-reactive protein (CRP). At the same time, an increase in adiponectin levels was observed, with the expression level of the adiponectin receptor 1 also being elevated. This fact can be related to peroxisome proliferator-activated receptor gamma coactivator 1-α (PGC-1α), peroxisome proliferator-activated receptor-α (PPAR-α), and AMP-activated protein kinase (pAMPK) levels being risen [103,104].

In another study, TNF-α and CRP levels in the serum of rats were increased by CP administration. However, following the orally delivered treatment with 300 mg RJ per day for two weeks, as well as the dosage of CP, this increase in both parameters was no longer visible [19,103]. Thus, RJ has been once again proven to possess anti-inflammatory capacity. Moreover, a similar outcome was observed regarding bronchoalveolar lavage fluid TNF-α levels of bleomycin-treated rats. In this investigation, 50 and 100 mg/kg of RJ was first administered for a week, orally. After that, a single intratracheal instillation of bleomycin (7.5 IU/kg) was applied. The researchers observed that RJ managed to reverse the effect of bleomycin on TNF-α levels in the bronchoalveolar lavage fluid and it also increased the IFN-γ levels in the fluid, which were previously decreased following the bleomycin treatment. Remarkably, the histopathological alterations caused by the toxic agent were also reversed by RJ [19,105].

Kohno et al. (2004) [56] took a closer look at the inflammation-reducing properties of RJ, at the cytokine level, uncovering the fact that the bee-derived secretion significantly reduces the levels of pro-inflammatory cytokines, such as TNF-α, IL-1, and IL-6 in murine peritoneal macrophages in vitro, in a dose-dependent fashion, without exerting any cytotoxicity on the cells [56]. Importantly, it has also been pointed out that RJ could play a significant role in improving the quality of life in the context of various autoimmune diseases, such as rheumatoid arthritis, due to its anti-inflammatory effect [12]. In a similar way, enzyme-treated RJ has the prospect of being developed as a nutraceutical, in order to improve the condition of patients suffering from inflammatory diseases, and even to prevent the occurrence of these diseases [5,106]. By the same token, RJ’s anti-inflammatory activity can improve the health status of rats affected by acetic acid-induced colitis and can enhance cell regeneration in the colon of the animals. The mechanism behind this effect is attributed to an enhanced mucin secretion by the colon cells and the improvement in their antioxidant status. Another mechanism could be represented by lowered levels of inflammation in the intestinal mucosa, due to a decreased number of CD68^+^ cells in the RJ-treated group [107].

Moreover, due to the anti-inflammatory response it brings into play, RJ can relieve muscle lipotoxicity and resistance to insulin in old age rats suffering from obesity, by reducing tumor necrosis factor-1 (TNF-1) adipose tissue and serum levels. In addition, laboratory animals in this condition could benefit from dietary supplementation with RJ not only in terms of the functioning of skeletal muscles but also through its impact on metabolism [106].

Yet another experiment that proved the anti-inflammatory effects of RJ was conducted by Aslan and Aksoy in 2015. Here, ethylene glycol was added to the drinking water of laboratory rats, thus obtaining the calcium oxalate urolithiasis animal model. The scientists highlighted that through this model, renal damage, oxidative stress, and inflammation were produced by ethylene glycol, and RJ managed to alleviate these negative effects, proving to be useful in aiding the medical treatment of urolithiasis and preventing it from happening in the first place. This bioactive property is thought to be strongly linked to RJ’s antioxidant and antiradical mechanisms [106].

### 4.3. Antioxidant Effect

Oxidative stress induced by ROS causes the oxidative degradation of important biomolecules, and being correlated with the appearance of diverse pathological processes and chronic illnesses such as cancer, cardiovascular diseases, cerebrovascular diseases, diabetes, osteoporosis, and renal failure [4,108,109,110]. The numerous advantages of using antioxidants to alleviate and help treat these debilitating conditions have been highlighted by researchers. At the dawn of the 21st century, scientists began to show interest in the antioxidant potential of RJ. Ever since, researchers all over the world have focused on studying this special bee-derived substance, uncovering its therapeutic potential [4].

Free radicals derived from oxygen, nitrogen, and sulfur have non-participating electrons in their structure, which is why they tend to react with other molecules, trying to take electrons from them, destabilizing and degrading them. These radicals are part of the group of molecules known as ROS and include superoxide anion, perhydroxyl radical, hydroxyl radical, hypochlorous acid, and hydrogen peroxide. Some authors consider reactive nitrogen species (RNS) to be a subclass of ROS, which includes nitric oxide, nitrous oxide, peroxynitrite, nitroxyl anion, and peroxynitrous acid [111]. Antioxidants are chemical agents that neutralize and inactivate ROS by accepting or donating electrons, having a protective role against oxidative stress, by significantly delaying or even preventing oxidation reactions that degrade biomolecules and cell organelles [111].

A growing body of evidence suggests that RJ can fulfill the role of a free-radical scavenger [18]. To exemplify, a group of researchers from Taiwan have evaluated the antioxidant and radical scavenging properties of RJ samples gathered from larvae of different ages that were subsequently grafted in artificial queen cells for distinct periods of time. They uncovered the fact that RJ manifested radical-scavenging activity upon 1,1-diphenyl-2-picrylhydrazyl free radicals and inhibited the formation of superoxide and hydroxyl radicals. The authors also highlighted that the antioxidant activity of RJ was significantly influenced by initial larval age and harvest time, the sample with the highest such activity being collected from the youngest, 1-day-old larvae at 24 h after larval grafting [112].

In another research carried out by Guo et al. in 2009, RJ proteins were hydrolyzed using protease N. The resulting peptides were found to hold strong antioxidant properties, which were evaluated regarding several processes such as hydroxyl, superoxide, and hydrogen peroxide radical-scavenging activities and chelating of metals. Out of all the tested peptides, 12 have been shown to scavenge hydroxyl radicals and three of them presented powerful scavenging ability for hydrogen peroxide, due to the ability of their hydroxyl group, which belongs to the phenolic residue, to donate its hydrogen atom. Nevertheless, no remarkable superoxide scavenging or metal-chelating effects were observed in this study [112].

Different in vivo models were used to explore the role of RJ in diminishing oxidative stress, by maintaining an adequate antioxidant status and protecting against ROS-induced toxicity. Silici et al. (2009) [113] investigated the role of RJ in the cases of nephrotoxicity and spermiotoxicity induced by cisplatin in rats. According to the authors, the antioxidant properties of RJ may be partially related to the presence of free amino acids, such as lysine, proline, cysteine, and aspartic acid. Alanine, glycine, valine, tyrosine, serine, cysteine, glutamic acid, threonine, glutamine, phenylalanine, leucine-isoleucine, and hydroxyproline are also found in RJ, but in smaller amounts. However, peptides that consist of two or three amino acids may show stronger anti-oxidative properties than the corresponding free amino acids [18].

Another research focused on the ability of RJ to attenuate the negative reaction of the organism when exposed to high doses of sodium fluoride, in a murine model. Aside from the radical-scavenging activity, the scientists believe that RJ’s antioxidant property could also be based on more indirect mechanisms, such as suppression of the expression of cytochrome P450 gene, known to be one of the main sources of intracellular superoxide, hydrogen peroxide, and hydroxyl free radicals. Another such mechanism is represented by hindering endogenous lipid peroxidation, via inhibiting the enzymes that catalyze these reactions [44]. Other mechanisms that have been attributed to the antioxidant effect of RJ are the modulating activity of retinol loss and the capacity to restore the accessibility of ascorbic acid (vitamin C) [114].

A recent investigation performed by Almeer et al. in 2019 [115] focused on examining the protective action of RJ on nephrotoxicity induced by cadmium intraperitoneal injection (6.5 mg/kg, for a week) in male mice, which were previously or subsequently treated with the yellowish-white secretion (85 mg/kg). The scientists managed to produce a murine model of kidney disease caused by oxidative stress, inflammation, and a deregulated apoptotic activity, manifested by both histopathological changes and alterations in various parameters (kidney injury molecule-1, nitric oxide, lipid peroxidation, metallothionein, glutathione (GSH), to name a few). RJ was able to normalize all the affected parameters in the pre-treated mice, protecting the kidneys against ROS and RNS-induced toxic effects. RJ’s capability to activate the nuclear factor erythroid 2-related factor 2/antioxidant responsive element (Nrf2/ARE) signaling pathway is thought to be the mechanism behind this nephroprotective effect [115].

Another recent study examined RJ’s in vivo hepatoprotective action in the case of nonalcoholic fatty liver disease. Oxidative stress in the hepatic cells plays a significant role in the pathophysiology of this condition [4]. An intragastric treatment with several doses of RJ; 150, 300, and 450 mg/kg to be specific, was applied to precedingly ovariectomized female rats (it is well known that menopause is one of the risk factors for nonalcoholic fatty liver disease). The RJ treatment was effective in alleviating hepatic steatosis, and improving the profile of fats in the serum, and the liver injury in ovariectomized rats was significantly diminished. All these positive effects occurred due to the antioxidant effect of RJ and can be attributed to diverse biological mechanisms, among which the most important may be the capacity of RJ to increase the enzymatic activity of antioxidant enzymes specific to the liver, therefore reducing the levels of oxidative stress. The author’s discovery indicates that a potential natural treatment alternative for this liver disorder can be represented by RJ, by means of its strong antioxidant effect [116].

Furthermore, RJ’s antioxidant potential may be used to prevent and possibly even treat certain illnesses either degenerative or chronic [33]. There are a few studies that back up this hypothesis. For instance, it has been shown that enzyme-treated RJ is effective in increasing the enzymatic activity of superoxide dismutase (SOD) and the amount of GSH, as well as in reducing intracellular ROS and nitric oxide produced in peritoneal macrophages obtained from BALB/c mice and treated with LPS. This experiment proves the fact that RJ hydrolysates have antioxidant activity. Moreover, it demonstrated that enzyme-treated RJ has surpassed non-treated RJ in terms of antioxidant activity [5]. The fact could be, at least partially, explained by the increase in bioavailability of RJ treated with commercial proteases. After the enzymatic treatment, it is certain that the content of free amino acids, as well as peptides with chains of distinct lengths, should increase, and their antioxidant properties are well-known. Additionally, the rise in GSH levels could be connected to the high quantity of cysteine and cystine that arises post-treatment [18].

Another example is represented by the research carried out by Park et al. in 2019 [60]. The researchers describe the antioxidant potential of MRJP2, highlighting its capability to protect DNA from the negative effects of ROS. Moreover, direct cell protection in opposition to ROS-induced stress was observed, by increasing cellular viability and decreasing levels of apoptosis induced by oxidative stress, as well as decreasing caspase-3 amounts, thus lowering its functional capacity.

There are also some additional in vitro experiments that take RJ’s antioxidative action into the discussion. Eshtiyaghi et al. (2016) [117] revealed that the RJ treatment of ovine oocytes improved their maturation via a positive influence of the cellular redox status. More specifically, an increase in SOD expression, cytosolic GSH levels and in the expression of mRNA responsible for glutathione peroxidase (GPx) synthesis was observed after a dose of 10 mg/mL of the interest substance was applied to the cells. Nevertheless, catalase (CAT) levels were not modulated. In contrast, Filipič et al. (2015) [99] seem to have demonstrated quite the opposite. They used the CaCo-2 cell line, represented by human colorectal adenocarcinoma, and explored the effects of RJ, 10-HDA and human interferon-alpha (HuIFN-αN3) on two parameters linked to oxidative stress: malondialdehyde (MDA) and GSH. By treating the cells with RJ accompanied by HuIFN-αN3 in a 2:1 proportion and RJ with 10-HDA also in a 2:1 ratio, the researchers obtained the following results. GSH cytosolic content was decreased, and MDA quantity was increased, suggesting higher extents of lipid peroxidation. The combination-based treatments have also been shown to be antiproliferative, not only because of the induction of cytotoxicity and apoptosis, but also due to the modulation of the antioxidative-prooxidative status [18]. Interestingly, this study managed to find a link between the anticancer and antioxidant effects of RJ.

Inoue et al. (2018) [118] looked into the activity of RJ and its fatty acid derivative, HPO-DAEE on the SH-SY5Y human neuroblastoma cell line. The scientists revealed that therapy with these interesting substances applied to the cells resulted in an increase in the synthesis of heme oxygenase-1 (HO-1) antioxidant enzymes. Thus, the treatment had a protective effect, reducing ROS-induced apoptosis and aiding the cells in acquiring oxidative stress resistance.

Additional substances that were used in order to induce oxidative stress in animal models were other chemotherapeutic drugs: methotrexate, taxol, paclitaxel, bleomycin; an artificial analogue for androgens, called oxymetholone; carbon tetrachloride, which is a solvent used in industry; and one immunosuppressive known as azathioprine. The antioxidant property of RJ manifested itself by bringing about positive outcomes in various representative parameters in all the studies concerning these substances [18].

## 5. The Apitherapeutic Potential of Royal Jelly

### 5.1. RJ as a Nutraceutical

Over the centuries, apitherapy and traditional Chinese medicine have promoted RJ and its health-supporting benefits. Since then, RJ has become a staple on the international market, being commercialized as ampoules, capsules, or tablets. Thus, the yellowish-white secretion plays an important role in the industry of nutraceuticals [4]. RJ-fortified skimmed milk [119] and milk with added RJ [120] are two examples of such nutraceutical products.

Functional foods can be part of a nutritional intervention, boosting overall health and complementing a healthy diet. Because of its highly nutritive composition, RJ can increase the nourishing quality of a plethora of foods [121]. Additionally, it can play a significant role in helping to prevent diseases such as hypertension, diabetes, and even, cancer [119].

Finally, the food of the queens could also be used as a nutritional supplement. This usefulness of RJ can be attributed to the bioactive compounds in its structure, namely the fatty acids, proteins, peptides, and phenolic compounds, more specifically, flavonoids [4]. Various supplement formulations were evaluated and the beneficial effects were proven. Chiu et al. (2017) [122] showed that capsules containing 0.35 g RJ exerted hypolipidemic effects and managed to reduce seric cholesterol and low-density lipoprotein cholesterol in human patients. Sharif and Darsareh [80] showed that capsules containing 1 g RJ were successful in diminishing the symptoms of menopause. The novel clinical trials that involved the administration of RJ are listed in Table 4.

In terms of RJ dosage, studies have shown that 100 mg/kg is generally the most effective, higher doses are seldom needed. For infants, 0.5 g/day is recommended and can be taken for 2 to 12 months. For children of 1–5 years old, a dose of 0.5 g/day RJ is suitable, while children of 5–12 years old can take 0.5–1 g/day. For adults, doses of 1–5 g/day can be administered, depending on the health problem. A higher dosage of 10 g/day can be taken for a briefer period of time (3 months, 10 days/month), in order to obtain stronger, faster results.

### 5.2. Wound Healing

Pasupuleti et al. (2017) [15] postulated that RJ can aid wound healing and this fact was proven by in vitro as well as in vivo experiments. Chronic wounds have turned out to be a challenge for clinicians over the decades. An investigation performed on the HaCaT cell line (human epidermal keratinocytes) focused on the proliferation and migration of the cells treated with RJ protein fractions soluble in water, in an in vitro scratch wound model. One particular fraction, which contained MRJP2, MRJP3, and MRJP7, possessed the capacity of accelerating wound healing, suggesting that these proteins can constitute a novel medication for solving this health problem. On the other hand, new research (in vivo studies in particular) is needed on the mechanisms underlying this rather complex process of wound healing, which are not yet fully understood [62]. The carboxyl-terminal pentapeptide repeats found in MRJP3 represent another potential wound-healing enhancer, incorporating functional groups with basic character. These groups can stimulate the proliferation of the Vero and THP-1 cell lines [131].

### 5.3. Aging and Longevity

It is well documented that RJ has important roles in the fertility and longevity of bees. The queen bees can lay a very large number of eggs every day (approximately 3000), and their lifespan is significantly longer than that of worker bees. Queens can live up to 5 years, while workers live only 45 days [32]. Regarding human cell lines, Jiang et al. (2018) claimed that mixtures of MRJPs of different concentrations (0.1–0.3 mg/mL) increased telomere length, decreased senescence, and stimulated proliferation in the cell line called HFL1 (human embryonic lung fibroblasts), by downregulating p53, catenin beta like-1, and the mammalian target of rapamycin (mTOR) [67].

Aging represents a physiological mechanism connected to the progressive decline of the organism [132]. The lipid fraction of RJ was proven to slow aging in human cells in culture. The two mechanisms responsible for this effect could be the inhibition of insulin-like growth factors synthesis and the stimulation of epidermal growth factor cellular signaling [32].

RJ is thought to influence longevity in humans due to positive effects on general health. These effects could be attributed to the anti-inflammatory and antioxidant activities of RJ, the consequence of which is the improvement of lipid profiles, oxidative status, glycemic status, and thus, the lower probability of developing metabolic diseases that can affect longevity [15,133]. Moreover, RJ can prolong lifespan by modulating insulin signaling. A peptide with insulin-like functions has been found in the interest substance. The decline in the synthesis of sexual hormones renders the body prone to aging more easily. These hormones are also known as the „markers of longevity”. It has been brought to light that RJ regulates sex hormones and its estrogen-like activity can be partially responsible for that [32]. Thus, it has been made clear that the bioactive compounds of RJ, among which amino acids, royalactin, MRJPs, acetylcholine, and 10-HDA can be listed, can influence the intricate process of aging. However, the exact mechanisms are not yet fully understood [32]. Of course, new studies are needed on this topical issue, in order to broaden the horizon of knowledge in the field.

### 5.4. Side Effects of Royal Jelly Administration

All over the globe, RJ is used in food supplements, beverages, cosmetics, and various other products. Although it is considered to be relatively safe and non-toxic [134], the side effects of RJ intake have been noticed and the majority of them are represented by allergic reactions, the symptoms of which can be ranked from mild to severe [135]. These manifestations include: skin rashes, contact dermatitis, allergic rhinitis, eczema, conjunctivitis, light gastrointestinal problems, hemorrhagic colitis, acute asthma, bronchospasm, anaphylactic shock, and in some cases, death [12,134,135].

Other products of the hive, such as honey, pollen, and venom, can cause less severe allergic reactions when compared to RJ. Therefore, persons that are allergic to these products should not consider oral administration of RJ. On top of that, caution is advised when recommending RJ to pregnant or breastfeeding women, children [12], and patients that have a history of allergic diseases, such as atopic dermatitis, asthma, or rhinitis. When allergic responses occur after consuming RJ for the first time, it may be due to the fact that some allergens are cross-reactive with RJ [135].

In order to establish the effective and safe use of RJ as a food ingredient, supplement, or nutraceutical agent, allergy tests should be run before taking RJ ingestion into consideration [12,135].

## 6. Conclusions and Future Perspectives

The beneficial properties of RJ were demonstrated by all the aforementioned cell culture, animal, and clinical studies, confirming the significant therapeutic potential of the food of the queens in diseases and its adjuvant effects on general health, as well as the possibility for it to become part of certain medicines in the future. In other words, RJ and its bioactive compounds could revolutionize alternative medicine, emerging as potential apitherapeutic products in various illnesses.

A further understanding of RJ’s mechanisms of action is required in order to move science forward in this field. This can only be achieved by filling the gaps in the existing research and considering the current perspectives. In this direction, scientists should focus on combination treatments involving RJ, the bioactive potential of RJ in human subjects, clinical studies with a larger number of patients, and the heterogeneity of RJ samples should also be taken into account. In the future, research should focus more on developing different RJ-based products (or products that contain biologically active compounds from RJ, such as MRJPs and 10-HDA) and their valorization on a practical level, in clinics, facilitating the transition of their use from bench to bedside. As it was described in this paper, from the whole set of MRJPs, the first four ones are the most studied, because they are found in the highest amounts. Further work should consider isolating and purifying MRJPs that are less studied, such as MRJPs 5–9 and investigating their properties. Other bioactive compounds of RJ that deserve to be researched more in-depth in the future are RJ-specific hormones and flavonoids and 10-HDA. Furthermore, there may still be some compounds in RJ that have not been yet discovered, thus, experiments in this sense seem to be advisable.

In this review, research on the antitumor, anti-inflammatory, and antioxidant functional properties of RJ was summarized. Therefore, the following subjects have been covered: chemical composition of RJ, quantitative determination, aspects related to storage of the interest substance, main bioactive compounds in RJ, a comprehensive review of three effects of RJ and three apitherapeutic applications of RJ in aging, longevity, wound healing, and in the nutraceutical industry.

In conclusion, RJ can be part of a nutritional intervention, complementing a healthy lifestyle. Together with proper medication, it can help relieve the symptoms of different illnesses and improve quality of life, through its powerful antioxidant, anti-inflammatory, and anticancer activities.

## Figures and Tables

**Table 1:** Nutritional requirements for quality assurance of RJ.

Compound	Method of Determination	Levels [%]	References
Water	Refractometric analysis	min 62.0–max 68.5	[32,33,34]
Lipids	Soxhlet method	min 2–max 8	[32,35,36]
10-HDA	High-performance liquid chromatography (HPLC); ultra-performance liquid chromatography, gas chromatography–mass spectrometry (GC/MS); NIR spectroscopy combined with chemometric methods	min 1.4	[4,23,32,34,37]
Proteins (total protein/soluble protein fraction)	Bradford assay; bicinchoninic acid (BCA) method	min 11–max 18	[32,34]
	Nitrogen content using carbon/hydrogen/nitrogen analyzer TruSpec (LECO, Saint Joseph, MI, USA)		[38]
Total carbohydrates	colorimetric method	min 7–max 18	[9,32,34]
Individual carbohydrates	Enzymatic method; HPLC method; GC method; Ionic chromatography; Infrared spectroscopy; Nuclear magnetic resonance	Fructose (2.3–7.6), glucose (2.9–8.1), sucrose (<0.1–2.1), maltose and maltotriose (0.0–1.0)	[9,34,39]

**Table 2:** Proximate composition of fresh RJ.

Compound	Levels [mg/100 g]	References
Minerals		
Macroelements		[40,41]
Na	0.3–13.8	
K	321.1–357.4	
Ca	22.8–24.0	
Mg	44.0–50.4	
P	338.4–412.1	
S	153.2–169.3	
Microelements		
Fe	n.d.	
Mn	0.01–0.08	
Zn	2.07–2.58	
Cr	0.03–0.15	
Cu	0.31–0.39	
**Vitamins**		
Vitamin A	1.10	[4,42]
Vitamin B1	2.06	
Vitamin B2	2.77	
Niacin (B3)	42.42	
Vitamin B5 (Pantothenic acid)	52.80	
Vitamin B6	11.90	
Vitamin B9 (Folic acid)	0.40	
Vitamin B12	0.15	
Vitamin C (Ascorbic acid)	2.00	
Vitamin D	0.2	
Vitamin E	5.00	
**Flavonoids**		
Quercetin	16.13	[43]
Naringin	0.47	
Hesperetin	0.85	
Galangin	0.51	
**Phenolic Acids**		
Chlorogenic Acid	37.61	[43]
Caffeic Acid	5.14	
Ferulic Acid	68.42	

**Table 3:** MRJP 1-9 brief description.

MRJP Member	Alternative Name	Amino Acids Number	Expression	Function	Reference
MRJP1	Royalactin, apalbumin 1, D III	413	Upregulated in nurse bee’s head compared to foragers; Upregulated in mated queen spermathecal fluid and spermatheca of the mated queen	Essential amino acids absorption; Source of energy for stored sperm; Worker honeybees’ temporal polytheism and phenotypic plasticity’s regulation; Social behavior; Learning; Memory	[53,55,56,57]
MRJP2	Apalbumin 2	435	Upregulated in nurse bee’s head compared to foragers	Nitrogen reserve; Worker honeybees temporal polytheism and phenotypic plasticity’s regulation	[56,58]
MRJP3	RJ protein RJP57-1	524	Upregulated in nurse bee’s head compared to foragers	Nitrogen supply; Worker honeybees temporal polytheism and phenotypic plasticity’s regulation	[55,56,57,58]
MRJP4	RJ protein RJP57-2	444	Upregulated in nurse bee’s head compared to foragers; Upregulated in mated queen spermathecal fluid and spermatheca of mated queen	Essential amino acids absorption; Source of energy for stored sperm; Worker honeybees temporal polytheism and phenotypic plasticity’s regulation	[53,55,56]
MRJP5		578	Upregulated in foragers’ head compared to nurses	Essential amino acids absorption; nitrogen reserve; Worker honeybees temporal polytheism and phenotypic plasticity’s regulation	[55,56]
MRJP6	Not applicable	417	Upregulated in foragers’ head compared to nurses; Upregulated in mated queen spermathecal fluid and spermatheca of the mated queen	Source of energy for stored sperm; Worker honeybees temporal polytheism and phenotypic plasticity’s regulation	[53,56]
MRJP7	Not applicable	426	Upregulated in nurse bee’s head compared to foragers	Worker honeybees’ temporal polytheism and phenotypic plasticity’s regulation	[56]
MRJP8	Not applicable	400	Evenly expressed in head, thorax, abdomen of all groups	Honeybee worker’s defense; queen’s long life span maintenance	[59,60]
MRJP9	Not applicable	403	Upregulated in virgin queens and workers bees	General philological role	[45]

**Table 4:** Novel clinical trials of RJ intake.

Target Condition	Dosage (mg/Day)	Duration	Participants Number	Gender	Study Type	Reference
Menopausal symptoms	1000 mg	8 weeks	200	Women	Double-blind	[79]
Sub-fertility	5000 mg	8 weeks	27	Men	Non-randomized	[123]
Type 2 diabetes mellitus	1000 mg	8 weeks	50	Men, women	Double-blind randomized	[124]
Hemodialysis	3600 mg	24 months	270	Men, women	Double-blind randomized	[125]
Multiple sclerosis	500 mg	3 weeks	100	Women	Double-blind randomized	[126]
Dry mouth sensation	800 mg	12 weeks	14	Men, women	Double-blind randomized	[127]
Traumatic brain injury	3000 mg	2 weeks	61	Men, women	Double-blind randomized	[128]
Infertility	100 mg	8 weeks	100	Men	Quasi-experimental	[129]
Genitourinary syndrome	1000 mg	8 weeks	192	Women	Double-blind randomized	[130]
Metastatic renal cell carcinoma	900 mg	13 weeks	33	Men, women	Double-blind randomized	[94]

## Data Availability

Not applicable.
